# Autoimmune Pancreatitis Type 2, Biliary Cysts and *Giardia lamblia*

**DOI:** 10.3390/children11091075

**Published:** 2024-09-01

**Authors:** Tonka Blažević, Mirna Natalija Aničić, Stanko Ćavar, Jurica Vuković

**Affiliations:** 1 Department of Pediatrics, General Hospital Zadar, BožePeričića5, 23000 Zadar, Croatia; 2 Department of Pediatrics, School of Medicine, University Hospital Centre Zagreb, University of Zagreb, Kišpatićeva12, 10000 Zagreb, Croatia; 3 Department of Surgery, School of Medicine, University Hospital Centre Zagreb, University of Zagreb, Kišpatićeva12, 10000 Zagreb, Croatia

**Keywords:** autoimmune pancreatitis, biliary cyst, *Giardia*

## Abstract

Autoimmune pancreatitis type 2 is a relatively novel entity with some still controversial issues. The current diagnostic algorithm relies on imaging studies and histology. Therapy includes corticosteroids with consequently low risk of relapse in the following year. However, the pathogenesis remains unclear, and data are insufficient for long-term prognosis. We have treated a 17-year-old boy whose autoimmune pancreatitis type 2 was revealed during surgery for a pre-existing biliary tract anomaly with concurrent protozoal infection. We discuss the co-occurrence of these conditions in terms of eventual pathogenesis correlation and combined effect on long-term prognosis.

## 1. Introduction

We report a case of autoimmune pancreatitis type 2 (AIP2) with unusual additional findings. AIP2 is a rare disease first described in 2003 [1]. Children with AIP2 usually present with acute abdominal pain, jaundice, fatigue and/or weight loss [2]. Transabdominal ultrasound is used as first-line imaging, detecting hypoechoic parenchyma, pancreatic enlargements and a pancreatic mass with or without a dilatated common bile duct. Since these findings are not pathognomonic and there is no marker specific for AIP2, histology is recommended for definitive diagnosis [2]. AIP is characterized by inflammatory infiltrate including lymphocytes and plasma cells. The periductal pattern and neutrophil-rich nature of the infiltrate is specific for type 2 [1]. The classic symptom triad of biliary cysts includes abdominal pain, palpable abdominal mass and jaundice. Our patient, like 85% of pediatric patients, presented with two of these features [3]. Based on anatomy, there are five types of cysts with several subtypes [4]. Treatment is surgical (e.g., cyst excision, Roux-en-Y hepaticojejunostomy and liver transplantation) and depends on cyst type [3]. Pathogenesis, unusual manifestations, diagnostic criteria, complications and prognosis of AIP2 still present challenges. We hope our experience can contribute to their elucidation.

## 2. Case Report

### 2.1. Preadmission History and Test Results

Six weeks before referral to our hospital, a previously healthy 17-year-old male with an unremarkable family history started complaining of upper abdominal pain following late-night meals. Proton pump inhibitor (IPP) therapy was initiated but symptoms persisted at the second visit, 10 days later. Physical examination revealed epigastric and supraumbilical tenderness. Laboratory results showed moderate elevation of inflammatory markers (CRP 41.9 mg/L) but, otherwise, were unremarkable (Hb 162 g/L, PLT 384 × 10^9^/L, L 11.6 × 10^9^/L, urea 3.3 mmol/L, creatinine 69 μmol/L, AST 18 U/L, ALT 26 U/L, GGT 21 U/L, ALP 98 U/L and LDH 140 U/L). Continuation of IPP therapy was recommended and the patient became pain-free. Due to jaundice with dark urine and pale stools, he was examined at a local pediatric emergency room. A cholestatic pattern of liver enzymes with conjugated hyperbilirubinemia and slightly shortened prothrombin time were noted (AST 187 U/L, ALT 499 U/L, GGT 473 U/L, ALP 281 U/L, LDH 212 U/L, amylase 41 U/L, total bilirubin 141 μmol/L, conjugated bilirubin 59 μmol/L, PT 0.59, INR 1.26 and fibrinogen 5.5 g/L). Abdominal ultrasound revealed an enlarged pancreas and hepatomegaly with dilatation of intrahepatic and extrahepatic ducts. The gallbladder was distended but did not show signs of inflammation. Proximal choledochus measured 16 mm and the peripapillary portion 5 mm. Serology results for viral hepatitis were negative. At this time, the patient was transferred to our hospital. He had lost 7 kg.

### 2.2. Initial Therapy and Evaluation of Cholestasis

**Figure 1:**
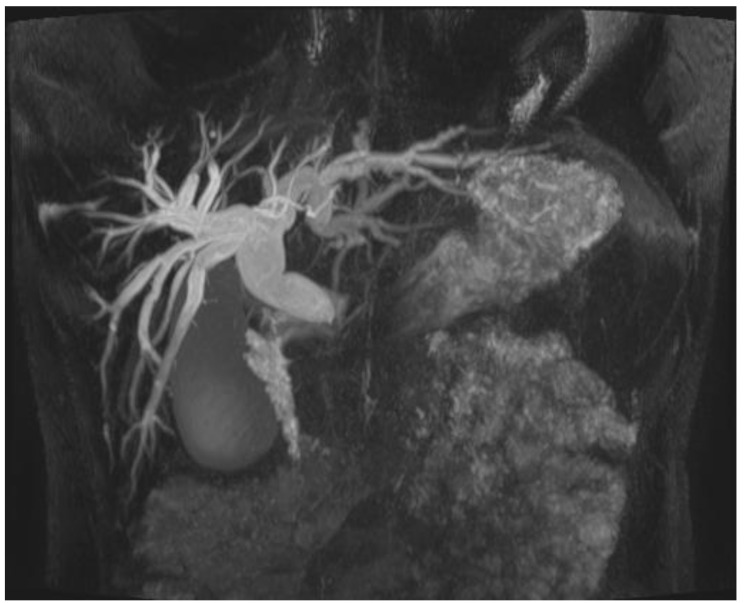
First MRCP.

According to previous findings, ursodeoxycholic acid and a spasmolytic were added to the therapy. Stools became normal. Liver enzymes, bilirubin and liver function tests were improving. Since other laboratory tests were inconclusive (Table 1), MRCP was performed to depict possible biliary obstruction, which revealed existence of fusiform extrahepatic ducts and intrahepatic biliary dilatation consistent with a Todani type Ic biliary cyst (Figure 1). The gallbladder was almost normal (89 mm, regular wall thickness), and the pancreas was homogenous with an enlarged head and narrow duct; no calcifications, capsule or fat changes were noted.

### 2.3. Pancreatitis and Other Additional Findings

During the scheduled choledochojejunostomy, a stiff and lobulated pancreas was noted. The surgeon performed a cholecystectomy and obtained pancreas samples by needle biopsy.

In the early postoperative period, while waiting for histology results, another unexpected finding occurred. Stool analysis confirmed the presence of *Giardia lamblia*. Metronidazole (3 × 250 mg, peroral) was administered for seven days. Histology of the pancreas showed fibrosis, acinar and ductal neutrophil infiltration, some mononuclear infiltration and low expression of IgG4 plasma cells, suggesting AIP2.

### 2.4. Treatment and Clinical Course

Fecal elastase results indicated exocrine insufficiency (23 μg/g, normal > 200 μg/g) and pancreatic enzyme replacement therapy was initiated concordantly with corticosteroids (prednisone 60 mg peroral). A second MRCP was performed10 days later to assess for therapy response. Compared with previous images, the pancreas was smaller and inhomogeneous; the pancreatic duct was still narrow, the intrahepatic ducts normalized and the extrahepatic ducts were half the initial size (10 mm) but the peripapillary part was slightly wider (2–3 mm in diameter, not visible before).

Also, several perihepatic collections were described. These were inhomogeneous, exhibiting diffusion restriction and post-contrast rim enhancement, and thus were characterized as abscesses. By CT-guided percutaneous transhepatic drainage, a small amount of hemorrhagic content was evacuated through a few days. Cytological analysis confirmed the specimen to be an inflamed hematoma. The day after the procedure, the patient became febrile with elevated inflammatory markers and was treated with three antimicrobial agents (ampicillin 4 × 2 g intravenous, meropenem 3 × 1.5 g intravenous and ciprofloxacin 2 × 500 mg peroral). After eight days, his condition resolved.

### 2.5. Second Surgery and Follow-Up

MRCP was performed a third time to confirm resolution of previous findings. Due to the persistently narrow peripapillary choledochus, the risk of recurrent biliary obstruction was deemed high and a choledochojejunostomy was scheduled in one month. In the meantime, fecal elastase results were improving and the patient gained some weight. Surgery was performed as scheduled with an unremarkable postoperative period.

Corticosteroid therapy was gradually tapered off during regular follow-up visits. The year after, therapy was gradually stopped with no complaints and former weight regained on a normal diet.

## 3. Discussion

### 3.1. Autoimmune Pancreatitis

Autoimmune pancreatitis (AIP) is a specific form of pancreatitis characterized by an excellent response to corticosteroids. Two different types of AIP are distinguished. Type 1 is the pancreatic manifestation of IgG4-related disease. It is marked by elevated serum IgG4 level and an IgG4/IgG ratio greater than 10%. Involvement of other organs (salivary glands, lacrimal glands, retroperitoneum, abdominal aorta, kidney, lungs and bile ducts) is not unusual. Histology shows lobule centric lymphoplasmacytic inflammation with diffuse infiltration of IgG4-positive plasma cells. Patients are typically over 40 years of age and male [1,5].

Type 2 is a less common type, constituting 10–15% of AIP. The estimated prevalence is 0.5/100,000 in the general population, with no significant difference between ethnic groups. Patients are children and young adults, and both sexes are equally affected. There is a wide spectrum of clinical presentations. Acute pancreatitis accounts for approximately 60% of cases and painless jaundice 30% [1]. Hepatic dysfunction, nonspecific abdominal symptoms and an incidental pancreatic mass have also been noted as dominant clinical features. Combinations of these with additional symptoms such as hyperglycemia or weight loss have also been described [6]. Because of the wide variety of symptoms, the diagnosis cannot be made on the basis of clinical presentation. No marker has been identified, as in the case of AIP type 1, but antineutrophil cytoplasmic antibodies and elevated CA 19-9 may be present [1]. Other laboratory results vary depending on the clinical presentation; even amylase and lipase are rarely informative, with results ranging from low to high. Imaging studies are more specific, showing diffuse or focal pancreatic enlargement, enhancement on late phase contrast images and occasionally irregular narrowing of the pancreatic duct or peripancreatic capsule. Although these findings reveal pancreatic disease in cases with a clinical presentation suggestive of involvement of other organs, they are not pathognomonic for AIP. Acute interstitial pancreatitis, drug-induced pancreatitis and number of malignancies must be considered before making the diagnosis of AIP2. In the presence of other organ involvement and elevated serum IgG4, diagnosis of type 1 can be made; otherwise, biopsy is essential [1]. In our patient, definitive diagnosis of AIP2 was based on criteria established by the International Association of Pancreatology, as pediatric criteria have not been proposed.

### 3.2. Biliary Cyst

Biliary cyst, formerly called choledochal cyst, is a congenital or acquired anomaly of the biliary tree. Todani et al. made the classification in 1977 based on the affected segment of the biliary tree. Types involving both extrahepatic and intrahepatic ducts are Ic and IVa [3]. In the revised classification, type Ic refers to a fusiform, diffuse, or cylindrical dilatation of choledochus, often extending continuously to the intrahepatic ducts [4]. It is usually associated with a pancreatobiliary junction (APBJ), which is a misconnection between the pancreatic duct and the choledochus outside the duodenal wall. This junction allows mixing of pancreatic juice and bile [4]. Reflux has been associated with biliary cysts, biliary tract carcinoma [7], pancreatitis and pseudopancreatitis (hyperamylasemia due to regurgitation of enzymes into the bloodstream through bile) [8]. Although biliary cysts are more frequent in the setting of APBJ, ductal stricture appears to be the key in pathogenesis of biliary cysts [4]. This theory is supported by cysts type Ib, II, III, IVb and V, which are rarely associated with APBJ. Furthermore, the morphology of the ductal stricture dictates the type of biliary dilatation. High-grade and long strictures are associated with cystic dilatation, in contrast to low-grade and short strictures, which cause fusiform dilatation. Similarly to type Ic, in type IVa, multiple cysts are present in both intrahepatic and extrahepatic bile ducts. The most important anatomic difference from type Icismultiple strictures, with obligatory hilar strictures causing intrahepatic cysts [4]. For that reason, the ductal anomaly in our patient does not meet criteria for Iva-type cyst.

### 3.3. Correlation between AIP and Biliary Cyst

In our patient, it is uncertain whether the ductal anomaly can be designated as a biliary cyst type Ic. There is no evidence of APBJ, but the initial ultrasound showed a normal distal choledochus (5 mm) with fusiform dilatation of the remaining bile ducts. Although the pancreas was enlarged, no compression was noted on the intrapancreatic portion of choledochus. Also, with fusiform dilatations of extrahepatic ducts, short, low-grade ductal stricture is expected, but MRCP demonstrated that pancreatitis caused long, high-grade stricture, which makes it possible that the biliary anomaly existed before AIP occurred. Whether it had any role in pathogenesis of the autoimmune process is unclear. Compression on the pancreas resulting in local obstruction of the small pancreatic ducts and release of enzymes would lead to exposure of pancreatic antigens. Such an event may be a starting point of AIP in a susceptible patient.

Even if pancreatitis and its tumefactive nature did not produce short, low-grade stricture of the choledochus, another potential mechanism has been documented. Sclerosing cholangitis with neutrophil infiltration in the epithelial layer, resembling the inflammation seen in AIP2, has been described in five children, one of which had concomitant pancreatitis [1]. This patient had an excellent response to prednisolone, suggesting that AIP2 may be a pancreatic manifestation of broader autoimmune disease. This disease, affecting the biliary tree, could have resulted in described choledochal pathology.

Therefore, co-occurrence of AIP and biliary dilatation requires careful investigation, especially because of long-term complications and prognosis. Dilatation of the common bile duct is common with AIP; sometimes it extends to the left or right hepatic duct or even the intrahepatic ducts [9,10]. In case of pre-existing biliary cysts, surgical procedure is an important part of treatment, not only to ensure bile drainage but also to prevent biliary malignancy. The risk of developing malignancy is almost 11% [11]. Even after the cyst is excised, it remains high. Therefore, long-term follow-up is recommended by yearly ultrasound studies and analysis of serum markers [3]. CA 19-9, CEA, IL-6, CYFRA 21-1, MMP-7, SSP411, miRNA and CTC can be used [12].

Previously, dilatation of bile ducts in the setting of AIP was often considered an indication for ERCP and bile duct stenting to ensure bile drainage through an enlarged pancreas [9,10,13,14]. We performed an MCRP instead. In comparison, ERCP is more precise at distinguishing pancreatic tumor from pancreatic focal enlargement and determining APBJ [15,16]. It also has therapeutic potential. However, it is not indicated routinely in AIP. The biliary tree is not affected in half the patients and, if pancreatitis is suspected (by signs other than a local mass), it gives limited additional information [9]. In our patient, it was probably even contraindicated [17] because surgery would still be necessary and pharmacotherapy proved efficient in bridging the waiting period with less risk for adverse events. In a recent large multicentric study, overall risk of ERCP adverse events was above 12%, with the occurrence of post-ERCP pancreatitis (PEP) in 8.5% patients. Bleeding, cardiopulmonary events, abdominal pain requiring hospitalization and perforation of the intestinal wall or biliary tract were also noted [10]. Before performing MRCP, we did not know our patient had AIP. If we had performed ERCP first, we could have misdiagnosed him as having PEP. Based on known risk factors for PEP (pancreatic indication, history of PEP, native major papilla, pancreatic duct cannulation, injection, stenting, stent removal, biliary duct stent removal, younger age, weight < 10 kg, APBJ, pancreas divisium, pancreatic stricture and sphincterotomy, and complexity), our patient with his unique pathology would be deemed as a high-risk patient [18,19]. Also, as percutaneous drainage intervention showed, he may be at high risk for other complications. Further, in case of suspicion of AIP, MRCP is recommended [2]. The American Society for Gastrointestinal Endoscopy suggest that ERCP is used for biliary cyst imaging if findings on cross-sectional imaging modalities such as MRCP and CT are equivocal [20].

### 3.4. Unexpected Guest

What makes this case even more interesting is another potential pathogenetic factor. *Giardia lamblia* is a wide-spread protozoa usually causing upper small intestine infection. It is known to spread to the biliary and pancreatic ducts and is associated with acute and chronic pancreatitis, pancreatic cancer [21], exocrine insufficiency [22], acalculous cholecystitis, cholangitis [23] and hepatic abscess [24]. There is a possibility that *Giardia lamblia* ascending from the intestine into the pancreas may have triggered the autoimmune process, leading to AIP. Furthermore, cholangitis associated with *Giardia* may have resulted in pathological formation of choledochus.

## 4. Conclusions

Interest in pediatric AIP has increased over the past two decades and a number of cases have been reported. Lately, the focus has been on diagnostic approach and criteria and not so much on ancillary findings. However, AIP2 has a low risk of relapse and concomitant pathology may determine long-term prognosis. Further research is needed to address this aspect, as there are not enough long-term studies to definitely characterize the clinical course of AIP as benign.

## Figures and Tables

**Table 1:** Initial laboratory results.

Variable	Value	Unit	Reference Range
E	5.47	×10^12^/L	4.43–5.88
Hb	158	g/L	129–166
L	6.9	×10^9^/L	4.4–11.6
PLT	308	×10^9^/L	178–420
ESR	12	mm/h	2–12
PT	0.85		>0.70
APTT	28.2	s	20.0–30.0
Fibrinogen	4.7	g/L	1.8–4.1
Bilirubin	134	μmol/L	7–30
Alkaline phosphatase	401	U/L	67–264
AST	181	U/L	11–38
ALT	551	U/L	10–33
GGT	490	U/L	23–91
Amylase	34	U/L	23–91
Lipase	48	U/L	13–60
Amylase (urine)	260	U/L	<400
Vitamin A	1.50	μmol/L	0.91–2.51
Vitamin E	20.2	μmol/L	21.8–50.4
C3	2.09	g/L	0.90–1.80
C4	0.30	g/L	0.10–0.40
IgE	170.0	kIU/L	<87
IgG	9.20	g/L	5.49–15.84
IgA	1.94	g/L	0.61–3.48
IgM	0.87	g/L	0.23–2.59
Proteins	76	g/L	66–81
Albumin	45.4	g/L	41.0–48.6
Alpha 1 globulin	4.1	g/L	2.1–3.6
Alpha 2 globulin	9.3	g/L	5.2–8.7
Beta globulin	9.0	g/L	6.2–9.6
Gamma globulin	8.2	g/L	8.2–13.8
Autoantibodies in AIH	Negative		
ANA (IFF)	Positive, AC-15		
Anti-PM-Scl	95	AU/mL	<30
Antibodies on parietal cells	positive	Titer	<1:20
Alpha 1 antitrypsin	>3.00	g/L	0.90–2.00
Ceruloplasmin	0.35	g/L	0.20–0.60
Ferritin	285.5	μg/L	15.0–200.0
Fecal calprotectin	131	μg/g	<50 negative, >180 IBD

## Data Availability

Data are included in the manuscript.
